# Enhancement of the EGSnrc code egs_chamber for fast fluence calculations of charged particles

**DOI:** 10.1016/j.zemedi.2022.04.003

**Published:** 2022-05-25

**Authors:** Thomas Failing, Günther H. Hartmann, Frank W. Hensley, Boris Keil, Klemens Zink

**Affiliations:** a Department for Radiotherapy and Radiooncology, University Medical Center Göttingen, Göttingen 37075, Germany; b Institute of Medical Physics and Radiation Protection (IMPS), University of Applied Sciences, Gießen 35390, Germany; c German Cancer Research Center (DKFZ), Heidelberg 69120, Germany; d Department for Radiotherapy and Radiooncology, University Medical Center Heidelberg, Heidelberg 69120, Germany; e Department for Radiotherapy and Radiooncology, University Medical Center Giessen-Marburg, Marburg 35043, Germany; f Marburg Iontherapy Center (MIT), Marburg 35043, Germany; g Diagnostic and Interventional Radiology, Philipps-University Marburg, Marburg 35043, Germany

**Keywords:** Monte Carlo simulations, EGSnrc, Variance reduction techniques, Charged particle fluence

## Abstract

**Purpose:**

Simulation of absorbed dose deposition in a detector is one of the key tasks of Monte Carlo (MC) dosimetry methodology. Recent publications (Hartmann and Zink, 2018; Hartmann and Zink, 2019; Hartmann et al., 2021) have shown that knowledge of the charged particle fluence differential in energy contributing to absorbed dose is useful to provide enhanced insight on how response depends on detector properties. While some EGSnrc MC codes provide output of charged particle spectra, they are often restricted in setup options or limited in calculation efficiency. For detector simulations, a promising approach is to upgrade the EGSnrc code egs_chamber which so far does not offer charged particle calculations.

**Methods:**

Since the user code cavity offers charged particle fluence calculation, the underlying algorithm was embedded in egs_chamber. The modified code was tested against two EGSnrc applications and DOSXYZnrc which was modified accordingly by one of the authors. Furthermore, the gain in efficiency achieved by photon cross section enhancement was determined quantitatively.

**Results:**

Electron and positron fluence spectra and restricted cema calculated by egs_chamber agreed well with the compared applications thus demonstrating the feasibility of the new code. Additionally, variance reduction techniques are now applicable also for fluence calculations. Depending on the simulation setup, considerable gains in efficiency were obtained by photon cross section enhancement.

**Conclusion:**

The enhanced egs_chamber code represents a valuable tool to investigate the response of detectors with respect to absorbed dose and fluence distribution and the perturbation caused by the detector in a reasonable computation time. By using intermediate phase space scoring, egs_chamber offers parallel calculation of charged particle fluence spectra for different detector configurations in one single run.

## 1 Introduction

Publication of the international IAEA Code of Practice TRS-398 [4] in 2000 turned out to be the basis for a considerable quality improvement in dosimetry for external radiotherapy [5]. Since that, a number of further developments have taken place in the field of dosimetry for external radiotherapy. An important advancement is due to Monte Carlo (MC) simulation of radiation transport which has become a widely used technique in the accurate calculation of dosimetric quantities for all beam types. Such quantities are stopping-power ratios, dose conversion factors, or perturbation correction factors required both for reference ionization chamber dosimetry and for dosimetry in non-reference conditions. A comprehensive review was recently published [6]. MC calculated data have indeed superseded many of the approximations used to determine the data in TRS-398 and are therefore explicitly considered in a forthcoming update of TRS-398. Additionally, MC calculated beam quality correction factors are already established in other dosimetry protocols such as the AAPM’s addendum to the TG-51 report [7] and the German DIN report 6800–2:2020–08 [8].

A key role is now taken by the MC calculated dose conversion factor which is defined as the ratio between the absorbed dose to water at a point of interest and the absorbed dose in the sensitive volume of a detector used for dosimetry. With this method, the factorization of the dose conversion factor into the stopping power ratio and one or more perturbation correction factors and hence the associated limitation to Bragg-Gray conditions can be avoided [1], [9]. A particularly appropriate tool to produce data for this approach is the MC code egs_chamber [10]. In order to reduce calculation time, a series of variance reduction techniques (VRTs) have been implemented in this user code. Additional to the VRTs photon splitting and Russian Roulette [11] which are already available in cavity, the photon cross section enhancement (XCSE) [12], [10] is implemented. In XCSE, free electrons, positrons or scattered photons are induced along a photon’s trajectory corresponding to increased reaction cross sections, while the original photon stays on its original track. To preserve the conservation of energy the generated particles have a reduced statistical weight. In addition, the methods of intermediate phase-space scoring (IPSS) and correlated sampling (CS) are included which allow the simulation of more than one geometry at the same time.

Another advantage of using MC simulations is to enable a closer look at the role of the charged particles, i.e. both primary and secondary electrons and positrons. There are a series of papers in which the fluence of charged particles differential in energy was recently considered in great detail [13], [14], [1], [15]. It particularly turned out that understanding how the charged particle fluence differential in energy is influenced by radiation conditions and by detector properties can considerably contribute to better understand the response characteristics of this detector [2].

Recent papers particularly address the dosimetrical quantity restricted cema (acronym of: converted energy per mass) which can serve as bridge between absorbed dose and the charged particle fluence [16], [3]. In these papers, a restricted cema based formalism for the determination of absorbed dose was suggested. For this purpose, but also for many other dosimetry related studies on the role of charged particles as listed above, there is an increasing use of detailed spectral fluence data. However, only a few MC codes provide these data. Examples are the codes FLURZnrc and cavity, both included in the EGSnrc system. Both codes have some limitations in application: the code FLURZnrc uses a cylindrical coordinate system which is not always appropriate to simulate a desired detector geometry while the code cavity is comparably inefficient for detector simulations. The code egs_chamber, on the other hand, better meets these requirements, however, it does not yet provide fast calculation of spectral charged particle fluence. The aim of this paper is to describe an enhancement of this code to become a general and at the same time fast MC calculation tool to provide data on absorbed dose and spectral fluence for various real detector and irradiation conditions. Results for calculated charged particle fluence, restricted cema as well as on the improved calculation efficiency are presented and compared with the results of existing MC codes for fluence calculations.

## 2 Theoretical background

### 2.1 Particle fluence

The quantity fluence, Φ, is given by Φ=dN/da, where dN is the number of particles incident on a sphere of cross-sectional area da. Thus, it refers to a mathematical point in a medium. However, it can be well approximated by the average fluence in a small volume which is assigned to that point. This approximation is most appropriate for MC simulations and therefore used in this work. The MC calculation of fluence within a volume is frequently based on a theorem of Kellerer [17], where the mean fluence is given by the sum of the track lengths of the particles, ds, per unit volume dV:

(1) $$\overset{¯}{\Phi }=\frac{\sum ds}{dV}$$

with unit cm^−2^. The quantity of interest in this work is the mean fluence differential in energy, ${\overset{¯}{\Phi }}_{E}$, with unit cm^−2^·MeV^−1^. The method based on Eq. (1) is used, for example, in the code FLURZnrc. A compilation of further methods appropriate for the implementation in MC codes is found in [15]. Since one of the methods was particularly useful for this work, it is also shortly described in the appendix.

### 2.2 Restricted cema

Based on the definition of ICRU Report 90 [18], the *restricted converted energy per mass* (cema) is calculated as:

(2) $$C_{\Delta }=\int_{\Delta }^{\infty }\Phi_{E}\frac{L_{\Delta }}{\rho }dE+{\mathrm{TE}}_{\Delta }$$

where $\Phi_{E}$ is the distribution of the total electron and positron fluence with respect to energy *E* at a point in a medium, $L_{\Delta }/\rho$ is the corresponding mass linear energy transfer and ${\mathrm{TE}}_{\Delta }$denotes the track end term [19], [18], [20] representing the sum of the kinetic energy of all electrons and positrons in the scoring volume with energies below the selected energy threshold value Δ. Restricted stopping power $S_{\Delta ,\mathrm{tot}}/\rho$ which is sometimes used in the definition of restricted cema is not exactly equal to linear energy transfer, however, at a value of Δ=10keV, the difference is negligible [20].

### 2.3 Determination of efficiency

The metric to quantify the efficiency ε of a Monte Carlo simulation can be determined as

(3) $$\varepsilon \propto \frac{1}{T\sigma^{2}}$$

using the total CPU hours *T* needed on a given computer to achieve a certain estimated statistical uncertainty σ. Since this is a general formalism, many quantities such as absorbed dose, fluence and restricted cema can be evaluated with that relationship. Any approach which decreases the time corresponding to a certain uncertainty or vice versa but does not injure the original result is called variance reduction technique.

## 3 Materials and Methods

### 3.1 Fluence calculations

In this work, four different MC codes of the EGSnrc system were applied for the calculation of the spectral fluence of charged particles and compared:

1. FLURZnrc[12]^1^
2. cavity[21]
3. a modified version of DOSXYZnrc [22], [1]
4. egs_chamber[10] as modified in this work

The first two codes are generally available, whereas the two modified codes represent in-house developments that would be ready for further distribution on request. Since cavity is not restricted to RZ-geometries and provides virtually all investigated quantities out of the box we consider it as the archetype of this work. The code DOSXYZnrc was originally developed for dose calculations in a rectilinear voxel system [22]. DOSXYZnrc was modified by one of the authors in order to offer more versatile applications, for instance for dose calculations in other volumes such as cylinders, spheres and also for many common detector types, or to permit fluence calculations in such volumes. Additional information on the method for fluence scoring is provided in [1], [15].

The modifications made to the user code egs_chamber in this work are briefly described next. Since cavity provides the full capability of fluence scoring and uncertainty estimation, the algorithm extracted from the corresponding section ausgab has been adopted. With this approach the built-in variance reduction techniques from egs_chamber such as photon cross section enhancement, Russian Roulette, intermediate phase space scoring and a region-based ECUT are conserved out of the box. However, the remaining VRT onegeom^2^ and correlated sampling are not provided.

### 3.2 Calculation of restricted cema

Using the data of the spectral electron and positron fluence obtained as described in the section above, the computation of restricted cema is performed by

(4) $$C_{\Delta }=\overset{i_{\max}}{\underset{i=i_{\Delta }}{\sum }}{\overset{¯}{\Phi }}_{E,i}{\left(\frac{S_{\Delta }}{\rho }\right)}_{i}dW+{\mathrm{TE}}_{\Delta }$$

where ${\overset{¯}{\Phi }}_{E,i}$ is the spectral fluence including liberated secondary charged particles, ${\left(S_{\Delta }/\rho \right)}_{i}$ is the restricted total mass stopping power at the energy of each bin center *i* beginning at the first bin above Δ (i.e. $i_{\Delta }$), dW is the energy corresponding to the bin width, and ${\mathrm{TE}}_{\Delta }$is the track end term representing the kinetic energy ($E_{\mathrm{kin}}$) of all charged particles with energies below the threshold value for particle tracking. The restricted total stopping power and restricted linear energy transfer are equal in a good approximation since their difference for Δ=10keV is negligible in our study [20], [23]. Based on the assumption that all energy contributions $E_{\mathrm{dep}}$ below Δ are deposited locally, the track end term could be therefore directly calculated [3] as:

(5) $${\mathrm{TE}}_{\Delta }=\sum E_{\mathrm{dep}}\left(E_{\mathrm{kin}}<\Delta \right)\text{.}$$

This formula was implemented in the modified versions of DOSXYZnrc and egs_chamber. However, this formula is usually not implemented in standard codes. Under the condition that a MC code provides the data of spectral fluence the restricted cema without track end term can be computed. If the absorbed dose is accessible too, the track end term can then be obtained due to the close agreement between absorbed dose *D* and restricted cema [16], [18] by:

(6) $${\mathrm{TE}}_{\Delta }\approx D-\overset{i_{\max}}{\underset{i=i_{\Delta }}{\sum }}{\overset{¯}{\Phi }}_{E,i}{\left(\frac{S_{\Delta }}{\rho }\right)}_{i}dW\text{.}$$

For all EGSnrc user codes, the calculation of the relative standard uncertainty of restricted cema was performed using the history-by-history statistical estimator method [24] as applied for the uncertainty determination of absorbed dose.

### 3.3 Test setups and comparison

**Figure 1:**
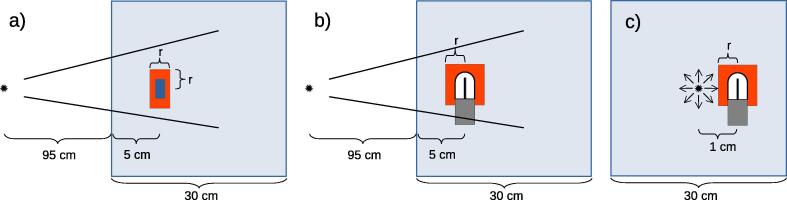
Schemes of the investigated beam setups. In all cases the XCSE water region in which XCSE is switched on in the egs_chamber simulations is marked by red cylinders with radius *r*. The water disc XCSE shell (shown in (a)) also varies the height in beam direction by the same. In case of an ionization chamber (shown in (b) and (c)) the height of the cylinder is fixed to 1.5 cm outstanding the active volume whereas the radius varies. Example (a): External point source collimated to a 4×4 cm^2^ square field size at 100 cm distance to the detector. The detector (here: blue water disc) is placed at 5 cm depth in a 30×30×30 cm^3^ cubic water phantom. Example (b): Same as (a) but with different detector such as PTW 31014 ionization chamber. Example c): Immersed point source located at the center of a 30×30×30 cm^3^ water phantom. The detector’s reference point (here: PTW 31014 ionization chamber with its sensitive volume in white) was at the same spot translated one centimeter away from the source. An analog immersed-source setup replacing the ionization chamber by a water voxel and XCSE region is not explicitly shown. Drawings are not to scale. (For interpretation of the references to colour in this figure legend, the reader is referred to the web version of this article.)

Results obtained with the modified user code egs_chamber were compared with those from FLURZnrc (which was specifically designed for fluence computation), from a modified version of DOSXYZnrc, and from cavity. Results were obtained for fluence spectra of electrons and positrons, for restricted cema, and for data on efficiency. Fig. 1 shows the investigated test setups. Two external radiation setups were simulated for a collimated point source geometry at SSD = 95 cm. As external sources the 6 MV Mohan spectrum [25] and the Co-60 Mora spectrum [26] were used. The third setup consisted of a point source emitting the spectrum of an Ir-192 microSelectron v2 HDR source immersed at the center of the water phantom. Results were scored in a small water disc of 0.1 cm radius and height or in the sensitive volume of a fully modelled PTW 31014 ionization chamber. Table 1 shows the chosen EGSnrc Monte Carlo parameters and simulation setups.

**Table 1:** Summary of the EGSnrc simulation parameters as used in this study. According to good practice the recommendations of AAPM report TG-268 [27] were followed.

Item name	Description	References
Code, version	EGSnrc (v2019a), egs_chamber, cavity, FLURZnrc, DOSXYZnrc, egs++ geometry package	[28], [22], [21]
Validation	—	[29]
Hardware & timing	12 cores, Intel® Xeon® CPU E5-2620 0 @ 2.00 GHz, 0.1 to 1000 total CPU hours	
Source description	Tabulated point sources, either collimated to a $4\times 4\;{\mathrm{cm}}^{2}$ square field size at SSD or isotropic	[25], [26]
Cross-sections	xcom photon cross sections. NIST ESTAR density effect corrections and mean excitation energies with ICRU 90 values where applicable. NIST Bremsstrahlung cross sections	[18], [30]
Transport parameters	ECUT = AE = 521 keV PCUT = AP = 10 keV Photon cross section = xcom Bound Compton Scattering = On/ norej Rayleigh scattering = On Atomic Relaxations = On/ eadl Brems cross sections = NIST Boundary crossing algorithm = exact Electron step algorithm = EGSnrc/ PRESTA-II ESTEPE = 0.25 All other parameters set to default	[28]
VRT	Varying XCSE factors from 0 to 2048 to determine optimum efficiency conditions	[10], [28]
Scored quantities	Absorbed dose, restricted cema, track end terms and charged particle fluence differential in energy to a water disc (radius and height 1 mm) or ionization chamber (PTW 31014). Detailed simulation setups are shown in Fig. 1.	
# histories/ statistical uncertainty	$10^{7}$ to $10^{11}$ histories depending on efficiency evaluations or benchmark simulations, uncertainty of 0.1 % for the latter (k=1)	
Statistical methods	History-by-history estimator	[24]
Post-processing	Collimated point source: Normalization per incident fluence, Isotropic point source: normalization per primary photon; De-noising of the spectra (6 MV Bremsstrahlung: averaging over 5 bins; otherwise over 2 bins), no filtering	

However, normalization of charged particle fluence computation is not performed identically in the investigated MC codes. One reason are different definitions of the incident primary photon spectrum: For a collimated point source FLURZnrc and DOSXYZnrc use the primary photon fluence per beam area (unit cm^2^) at the SSD. Because the beam area is cancelled out, the secondary charged particle fluence is then given in units of MeV^−1^. In cavity and egs_chamber the incident photon spectrum is referred to steradian and independent on the SSD. For comparison, all external beam radiation spectra were therefore normalized per incident photon fluence per MeV^−1^ independent of the SSD but normalized to the field size at reference depth. The immersed point source is normalized per initial particle in every user code thus the secondary charged particle spectra have units MeV^−1^cm^−2^. Regarding comparable examples as shown in Fig. 1 and no use of VRTs, all investigated user codes yield similar calculation efficiencies. Therefore all efficiency enhancements can be compared to the original cavity code. The variance reduction technique from egs_chamber used in this work was photon cross section enhancement. To investigate the resulting efficiency gain, the detectors were embedded in water shells of varying sizes with radii *r* in which XCSE was applied, as shown in Fig. 1. For the ionization chamber the water shells had a fixed length extending outside the active volume. For water disc cases both radii and height were extended by the same amount. Each simulation had one fixed XCSE value that was assigned to all detector parts including an extended stem and the surrounding shell.

It is noteworthy that the investigation of VR-techniques can become quite tedious due to the interference of the different techniques [31] or the dependency on radiation setups because several VRT parameters have to be considered together in every beam setup [10]. For this reason we investigated the efficiency gain due to XCSE only. Additionally, it is found in [31] that XCSE offers the greatest efficiency enhancement when the VRTs are considered individually. The aim at this point is to compare the charged particle fluence scoring techniques and the corresponding efficiency gain resulting from XCSE in egs_chamber systematically in a given scenario.

## 4 Results

### 4.1 Charged particle fluence spectra and restricted cema computation

As initial verification - and based on the same initial random numbers - the enhanced egs_chamber as well as the original egs_chamber and cavity user codes yield exactly the same results regarding all investigated quantities if no additional VRTs from egs_chamber are used. This is even the case for charged particle fluence data although not provided by the original egs_chamber user code.

**Figure 2:**
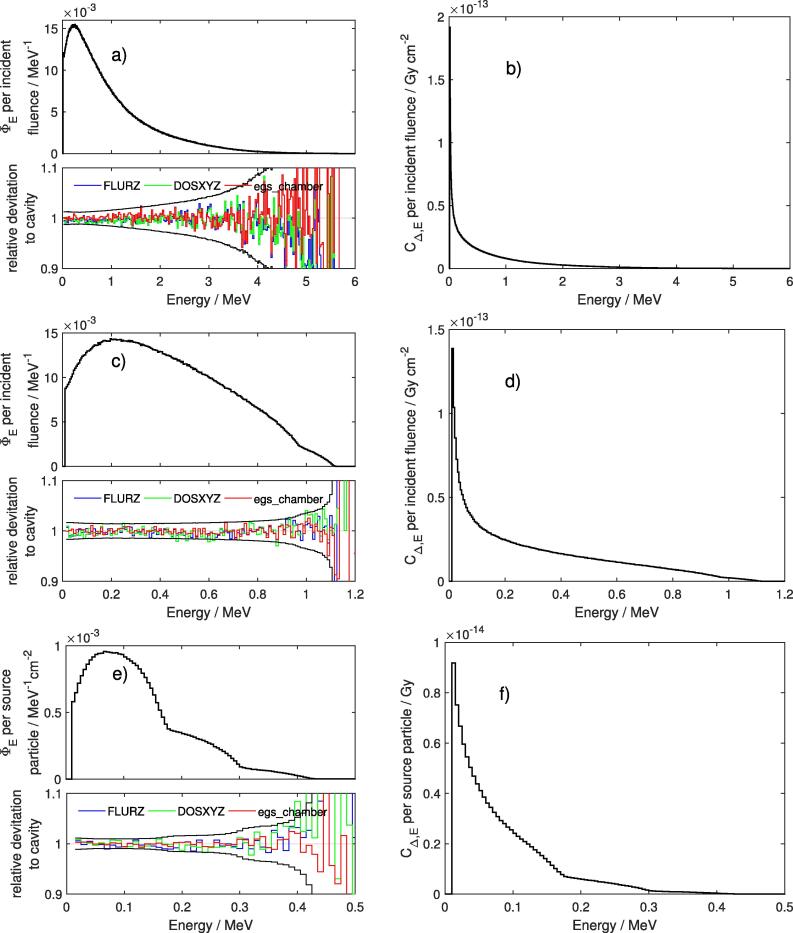
Electron fluence (left: (a), (c), (e)) and restricted cema (right: (b), (d), (f)) differential in energy calculated with cavity (bold black lines) with 5 keV bin width for an irradiation of a PTW 31014 ionization chamber with external-beam 6 MV Bremsstrahlung (top row), Co-60 (second row) or immersed-source Ir-192 photons (bottom row) as shown in Fig. 1(b) and (c). Due to the almost identical results, only the graphs of cavity are shown. The relative deviations of the results from other user codes in comparison to cavity are shown under the corresponding spectra with the mean standard deviations of the quotients in black.

Fig. 2 shows the electron spectra differential in energy and the corresponding restricted cema in the volume of interest obtained using the user code cavity and the corresponding relative deviations from egs_chamber, FLURZnrc and DOSXYZnrc. For the sake of clarity the spectra of secondary positrons differential in energy are not shown since they are at most responsible for only about 1 % of the response in a 6 MV Bremsstrahlung photon beam. The analyzed volume refers to the sensitive, air-filled cavity of the fully modelled ionization chamber, type PTW 31014.

All datasets belonging to one simulation setup agree with each other within their statistical uncertainties. Numerical deviations between cavity and the other user codes exceed 5 % only at high energies. For the line spectra of Co-60 and Ir-192 the corresponding Compton edges at about 1.1 MeV and 0.95 MeV or 0.42 MeV, 0.3 MeV and 0.18 MeV, respectively, are clearly visible in all fluence scoring algorithms. Contrary to the electron or positron fluence differential in energy, the peak values of the restricted cema differential in energy appear at the first bin above Δ (i.e. 10 keV). This observation can be explained by the strong energy dependence of the restricted stopping powers used for the computation of restricted cema which decrease monotonically at low energies.

Table 2 shows the results concerning absorbed dose, restricted cema and track end term for external irradiation with Co-60 photons as in Fig. 1(a). The normalization was performed independent of the SSD for a beam area of $4\times 4\;{\mathrm{cm}}^{2}$ at 100 cm distance to the source. Again the results agree within their statistical uncertainties.

**Table 2:** Absorbed dose *D*, restricted cema $C_{\Delta }$ (as calculated by Eq. (4) including ${\mathrm{TE}}_{\Delta }$as calculated by Eq. (5) and track end term ${\mathrm{TE}}_{\Delta }$ per incident photon fluence (i.e. in Gy cm^−2^) calculated for the irradiation of a water disc with Co-60 photons in the external beam setup from Fig. 1 a). The relative standard uncertainty of the final digit is shown in parentheses for each value.

	*D*	$C_{\Delta }$	${\mathrm{TE}}_{\Delta }$
egs_chamber	$3.78(1)\cdot 10^{-12}$	$3.78(1)\cdot 10^{-12}$	$0.280(1)\cdot 10^{-12}$
cavity	$3.78(1)\cdot 10^{-12}$	—	$0.280(3)\cdot 10^{-12}$
DOSXYZnrc	$3.77(2)\cdot 10^{-12}$	$3.77(2)\cdot 10^{-12}$	$0.279(2)\cdot 10^{-12}$
FLURZnrc	$3.79(3)\cdot 10^{-12}$	$3.79(3)\cdot 10^{-12}$	$0.275(4)\cdot 10^{-12}$

For radiation qualities of 6 MV Bremsstrahlung and higher energies the contributions from positrons to restricted cema exceed 1 % and are therefore not negligible. Additionally, the relative contributions from ${\mathrm{TE}}_{\Delta }$ are energy dependent and vary from 6% for 6 MV Bremsstrahlung up to 14 % for Ir-192.

### 4.2 Efficiency evaluation

**Figure 3:**
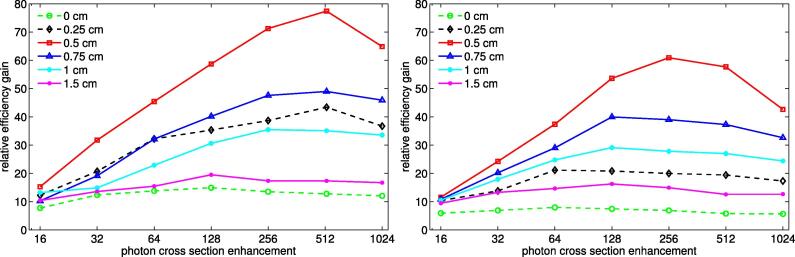
Relative efficiency gains in the calculations of absorbed dose (left) and restricted cema without ${\mathrm{TE}}_{\Delta }$ (right) using photon cross section enhancement with various shell thicknesses *r* compared to no use of VRTs. The investigated setup consisted of the PTW 31014 ionization chamber externally irradiated with a Co-60 point source as shown in Fig. 1(b). A shell thickness of 0 cm corresponds to a cross section enhancement of the ionization chamber parts only.

Fig. 3 shows the relative efficiency gain of egs_chamber simulations with various XCSE factors and shell thicknesses in the calculation of absorbed dose or restricted cema without track end term. The track end term was omitted in the efficiency evaluation because the restricted cema was calculated in the user code based on the charged particle fluence scoring algorithm which ignores particle energies below Δ. For these calculations a PTW 31014 ionization chamber was placed in a water phantom and externally irradiated with Co-60 photons as shown in Fig. 1(b). In this example the efficiencies first increase with increasing enhancement of the cross sections. This behavior reaches a maximum after which the computation time to trace the growing number of secondary charged particles initiated by the higher cross sections begins to increasingly reduce the efficiency.

The photon cross section enhancement shell thickness also has an impact on the simulation efficiency. Similar to an increased XCSE value, a thicker shell around the detector leads to more photon interactions causing an increase in charged particles scored in the detection volume. And again more interactions require more computation resources. Both with enhancement of the value of the cross sections and enhancement of the volume in which XCSE is applied, the relative efficiency gain depends on the computed quantity. In the example shown in Fig. 3 the maximum achievable relative efficiency gains concerning restricted cema without ${\mathrm{TE}}_{\Delta }$ and absorbed dose calculations are different and the efficiency peaks are achieved at different values of XCSE.

Table 3 shows the relative efficiency gain for all investigated beam setups at the optimum XCSE value and shell thickness. Several observations can be made:

**Table 3:** Relative efficiency gain in the calculation of absorbed dose and restricted cema without track end term with egs_chamber at the optimum values of photon cross section enhancement conditions compared to no use of VRTs. The parameter *r* depicts the surrounding XCSE shell where r=0 means an enhancement of the cross sections of only the components of the ionization chamber. The calculations were performed in the external beam setup for 6 MV Bremsstrahlung and Co-60 photons as shown in Fig. 1(a) and (b), and in the immersed-source setup for Ir-192 photons as shown in Fig. 1(c).

		absorbed dose			restricted cema without ${\mathrm{TE}}_{\Delta }$		
		XCSE	*r*/cm	rel. eff. gain	XCSE	*r*/ cm	rel. eff. gain
6 MV	water voxel	64	1	19.3	64	1	19.3
	PTW 31014	128	0.75	48.9	128	0.75	26.3
Co-60	water voxel	256	0.75	51.4	256	0.75	51.7
	PTW 31014	512	0.5	77.4	256	0.5	60.9
Ir-192	water voxel	512	0.25	89.2	512	0.25	92.6
	PTW 31014	1024	0	97.7	512	0	147.7

First, the relative efficiency gain depends on the radiation quality and increases with decreasing particle energies. Second, the optimum cross section enhancement value increases with decreasing particle energies whereas the optimum shell thickness shows an inverse behavior. Third, the efficiency improvement potential is larger in ionization chamber simulations in every investigated setup. Fourth, the optimum XCSE conditions for absorbed dose and restricted cema without ${\mathrm{TE}}_{\Delta }$ calculations are equal for water voxels. The parameters differ only slightly for the ionization chamber simulations where the setups referring to restricted cema without ${\mathrm{TE}}_{\Delta }$ tend to smaller enhancement values.

## 5 Discussion

As stated in the EGSnrc C++ class library [21] the track length based fluence scoring algorithm as used in FLURZnrc assumes the stopping powers to be constant along a charged particle step. This might affect spectra at regions where the progression of the stopping powers is steep. Our results show that this applies only to the first bin above Δ (i.e. 10 keV) in water disc simulations, which yields about 5 % more than the other algorithms in certain energy bins. One workaround is the reduction of the MC parameter ESTEPE so that the condensed history steps are small enough for an accurate scoring with constant stopping powers. This miscalculation on charged particle fluence scoring in FLURZnrc does not impair the resulting restricted cema since its impact is considerably smaller than the statistical uncertainty of restricted cema calculation. Thus the effect is negligible in our study and may be accounted for in the overall uncertainty budget.

The investigated VR-technique photon cross section enhancement is well established since several years in various EGSnrc user codes [11], [28], [12]. With the introduction of XCSE in egs_chamber, the application to charged particle fluence computations was explicitly mentioned in [10]. Furthermore the EGSnrc standard statistical uncertainty history-by-history estimator is also explicitly dedicated to arbitrary quantities such as fluence [24]. XCSE as used in egs_chamber offers a dramatic efficiency gain especially for small scoring regions compared to the irradiated volume [10]. Our results confirm this especially regarding an internal irradiation as shown in Fig. 1(c).

The relative efficiency gains between absorbed dose and restricted cema without ${\mathrm{TE}}_{\Delta }$ differ for ionization chamber simulations. Fluence scoring in cavity is done using track-length estimation by precomputed stopping powers from the EGSnrc database. Restricted cema can then be obtained as described in Section 3.2. Absorbed dose scoring tallies up the energy deposited due to ionization (continuous energy losses) and discrete interactions. As the energy of the charged particles changes, the scoring can be more or less efficient and hence the dose scoring may lead to a different statistical uncertainty than restricted cema computation for the same number of traced particles. A similar mechanism could occur in the calculation of kerma using photon fluence and mass-energy absorption coefficients in comparison to using the kerma approximation of depositing the energy of secondary charged particles on the spot.

**Figure 4:**
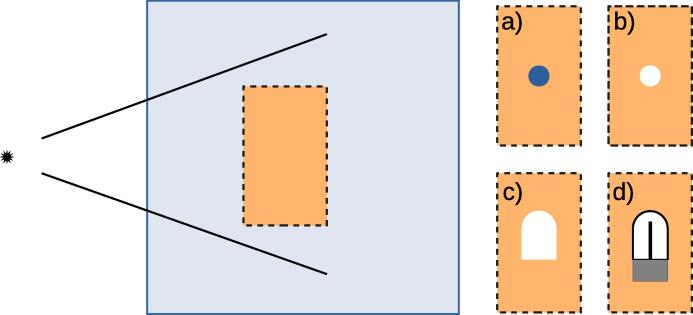
Practical example for the use of intermediate phase space scoring in an external beam setup. Particles, primary as well as secondary of nature, entering a specified region (orange dashed box) in the phantom are intermediately stopped and re-run several times from that point with different detectors inside. In this exemplary case the different detectors consist of (a) a water voxel, (b) an air voxel, (c) the bare air cavity volume and (d) the fully modelled thimble ionization chamber. With these different detector types it is possible to simulate perturbation factors as proposed in [15], [2], [16]. Drawings are not to scale. (For interpretation of the references to colour in this figure legend, the reader is referred to the web version of this article.)

By using other variance reduction techniques such as intermediate phase space scoring in the enhanced egs_chamber code the computation of several detector compositions needed for perturbation correction factors may be obtained in one single simulation run. Fig. 4 depicts one practical example of the combined simulation of the components related to the dose conversion factor for an ionization chamber split into stopping power ratio (Fig. 4(a) to (b)) and several perturbation correction factors (Fig. 4(b) to (c) and (c) to (d)). Applying this example to the setups as shown in Fig. 1 with external or immersed source and adjusted XCSE conditions for each detector, a further relative efficiency enhancement of 1.52, 1.57 and 1.61 for external 6 MV Bremsstrahlung, external Co-60 and immersed source Ir-192 photon beams could be observed respectively.

If the phase space scoring volume is large enough and contains all detector components in every position, several detector positions inside a phantom can be simulated simultaneously. An additional enhancement would be the implementation of correlated sampling [28], [21] for charged particle fluence scoring so that spectral resolved fluence or restricted cema quotients can be calculated.

## 6 Conclusions

This work shows a straightforward enhancement of egs_chamber to calculate charged particle fluence using methods already present in the EGSnrc framework and in particular in cavity. The EGSnrc applications are built modular so that there is not much interference with the inserted code. The comparison with several EGSnrc user codes show good agreement which proves that the implemented charged particle fluence scoring in egs_chamber does not impair the original code. Furthermore, in our calculations, the different radiation setups (external or immersed source) and the use of variance reduction techniques showed no influence on the charged particle fluence spectra.

Using VRT provided by EGSnrc such as XCSE an improved simulation efficiency could be observed. There are even more techniques to raise the efficiency implemented in egs_chamber such as a region-based ECUT, intermediate phase space scoring (IPSS), Correlated Sampling and onegeom. The latter allows several simulation geometries to be equal while using different scoring regions parallel. Region-based ECUT, XCSE and IPSS work out of the box whereas Correlated Sampling and onegeom require additional modifications.

With the given approach it is possible to achieve spectral information even for sophisticated demands within acceptable computing time. Additionally the restricted cema as a link between absorbed dose and charged particle fluence can also be calculated. Since the restricted cema methodology has the advantage of being applicable in setups which do not fulfill Bragg-Gray conditions [16] the enhanced egs_chamber code is well suited for further investigations on detector developments and non-reference conditions.

## Conflict of Interest

This work is part of a dissertation project of Technische Hochschule Mittelhessen University of Applied Sciences at the Graduate Center of Engineering Sciences at the Research Campus of Central Hessen.

The authors declare that they have no known competing financial interests or personal relationships that could have appeared to influence the work reported in this paper.

## Figures and Tables

**Figure 5:**
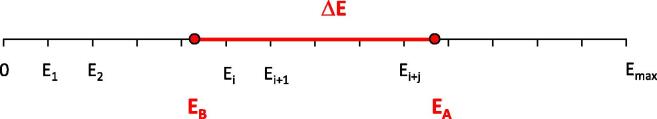
Diagram showing the energy deposition ΔE of an electron or positron in relation to a linear energy grid 0, E_1_, E_2_, … E_max_.

## Appendix A Calculation of spectral charged particle fluence

As stated in Eq. (1), the mean fluence of charged particles in a scoring volume can be derived from the sum of their path lengths per unit volume. In EGSnrc, an electron or positron path length is obtained either in the single scattering mode, i.e. between two scattering events, or in the condensed history mode, i.e. between two hard (inelastic) interaction sites. In both cases, the relation between a given path length to the energy loss over this path and vice versa must be known. For this relation, the ‘continuous slowing down approximation’ is assumed where the energy loss results from sub-threshold processes only. Thus, the energy loss ΔE of an electron or positron is formally linked to its corresponding path length *s* through:

(.1) $$\Delta E=\int_{0}^{s}L_{\Delta }(s^{\prime })ds^{\prime }$$

where $L_{\Delta }$ is the linear energy transfer, also called restricted stopping power. It follows that an electron or positron path can be characterized by three parameters of kinetic energy: The energy loss ΔE associated to the path length *s*, the energy $E_{A}$ at the path start, and $E_{B}=E_{A}-\Delta E$ at its stop. The position of this parameter with respect to a linear energy grid 0, E_1_, E_2_, … E_max_ is shown in Fig. 5.

The method of fluence calculation is now essentially based on two steps:

Step 1: Each energy deposition (equivalent with the energy loss ΔE) is split into a number of single subsections adjusted to the energy binning which will be used to present the electron or positron fluence differential in energy. Using the example of Fig. 5, the first subsection is E_*i*_-$E_{B}$, the intermediate subsections are identical to the bin width dW, and the last subsection is $E_{A}$-E_*i*+*j*_. The quotient of each subsection by ΔE is termed α.

Step 2: By means of the quotient $\alpha_{i,j}$ where the index *j* refers to a specific energy loss ΔE during the MC simulation, the spectral fluence ${\overset{¯}{\Phi }}_{E,i}$ at bin *i* within the scoring volume *V* can now directly be derived.

The two methods used in this work are:

1. track length based:
  $\;\Phi_{E,i}=\sum_{j=1}^{M}\frac{1}{V}\cdot \frac{\alpha_{i,j}}{dW}\cdot s_{j}$where *j* refers to one particle track out of the total number of histories *M*, and $s_{j}$ is the path length associated to the energy loss $\Delta E_{j}$; this method is implemented in FLURZnrc.
2. energy deposition based:
  $\;{\overset{¯}{\Phi }}_{E,i}=\sum_{j=1}^{M}\frac{1}{V}\cdot \frac{\alpha_{i,j}}{dW}\cdot \frac{\Delta E_{j}}{L_{\Delta ,i}}$where $L_{\Delta ,i}$ is the linear energy transfer of the material in the scoring volume at bin *i*. In the modified version of DOSXYZnrc, $L_{\Delta ,i}$ is taken at the energy of bin center, whereas in cavity, it is - according to the documentation - at the lower energy of each bin. Actually, however, it is also at bin center.

## Footnotes

2 This VRT allows several simulation geometries to be equal while using different scoring regions parallel. One useful application are Fano calculations.

## Footnotes

1 Since results on absorbed dose and restricted cema are usually not given with the standard version, this code was only slightly extended to provide such data as well. A publicly available alternative would be to use cavity with the charged particle scoring algorithm from FLURZnrc as stated in [21].
